# Changes in Body Composition in the Two Years after Initiation of Haemodialysis: A Retrospective Cohort Study

**DOI:** 10.3390/nu8110702

**Published:** 2016-11-04

**Authors:** David Keane, Claire Gardiner, Elizabeth Lindley, Simon Lines, Graham Woodrow, Mark Wright

**Affiliations:** 1 Department of Renal Medicine, Leeds Teaching Hospitals NHS Trust, Leeds LS97TF, UK; elizabeth.lindley@nhs.net (E.L.); graham.woodrow@nhs.net (G.W.); mark.wright8@nhs.net (M.W.); 2 Department of Medical Physics, Leeds Teaching Hospitals NHS Trust, Leeds LS97TF, UK; 3 NIHR Devices for Dignity Healthcare Technology Co-operative, Sheffield Teaching Hospitals Trust, Sheffield S102JF, UK; 4 Department of Nutrition and Dietetics, Leeds Teaching Hospitals NHS Trust, Leeds LS97TF, UK; claire.gardiner2@nhs.net; 5 Norfolk and Norwich University Hospitals NHS Foundation Trust, Norwich NR4 7UY, UK; simon.lines@nnuh.nhs.uk

**Keywords:** bioelectrical impedance, body composition, hemodialysis, nutrition assessment

## Abstract

Malnutrition is common in haemodialysis (HD) and is linked to poor outcomes. This study aimed to describe changes in body composition after the initiation of HD and investigate whether any routinely collected parameters were associated with these changes. The study cohort came from the HD population of a single centre between 2009 and 2014. Body composition measurements were obtained from a database of bioimpedance results using the Body Composition Monitor (BCM), while demographics and laboratory values came from the renal unit database. Primary outcomes were changes in normohydration weight, lean tissue mass and adipose tissue mass over the two years after HD initiation. A total of 299 patients were included in the primary analyses, showing an increase in adipose tissue, loss of lean tissue and no significant change in normohydration weight. None of the routinely collected parameters were associated with the lean tissue changes. Loss of lean tissue over the first year of dialysis was associated with increased mortality. The results showing loss of lean tissue that is not limited to those traditionally assumed to be at high risk supports interventions to maintain or improve lean tissue as soon as possible after the initiation of HD. It highlights the importance of monitoring nutrition and the potential for routine use of bioimpedance.

## 1. Introduction

Malnutrition is common in haemodialysis (HD) patients—reports suggest anywhere between 30% and 70% of patients are affected [1,2]—and it is strongly associated with mortality, inferior quality of life, hospitalisation rates and morbidity [3,4,5].

Losses of lean and adipose tissue are inherently linked to many common assessments of malnutrition—such as anthropometric measurements and malnutrition scoring tools that monitor changes in weight—and indirectly with other parameters used for assessment, such as albumin. In HD patients it is well established that lean tissue loss is independently associated with poor outcomes [6], but the effect of changes in fat mass is more complicated. There is good epidemiological evidence that body mass index (BMI) is associated with survival in HD patients across all BMI classifications, leading to the so called ‘obesity paradox’ whereby high BMI, typically seen as a risk factor, offers a survival benefit [7]. However, BMI is a measure of body size rather than fat mass and attempts to isolate the potential survival benefit of increased fat mass have shown mixed result [8,9]. It has been suggested that increased abdominal fat is associated with mortality [10] and there are well described deleterious effects associated with increased fat mass, such as insulin resistance, inflammation, dyslipidemia, atherosclerosis and coronary calcification [11].

Numerous routinely collected parameters have been linked to nutritional changes in HD. Malnourished patients identified both by subjective global assessment (SGA) and bioimpedance analysis were shown to have elevated C-reactive protein (CRP) compared to well-nourished peers [12,13]. There is evidence that under-dialysis is related to poor nutritional state [14]. However the hemodialysis (HEMO) study showed higher dialysis dose did not prevent deterioration of nutritional state when compared to the dose required to achieve dialysis adequacy standards [15], suggesting that the effect of dialysis dose on nutritional state may plateau. The presence of insulin resistance and increased co-morbidities in diabetic patients is associated with muscle wasting, but there is contrasting evidence about whether the prevalence of malnutrition is truly higher in diabetic as compared to non-diabetic HD patients [13,16,17]. As in the general population, it has been shown that fat mass increases and muscle mass decreases with age in HD patients [18]. Acidosis and abnormalities of insulin and insulin growth factor I (IGF-I) metabolism also contribute to muscle wasting [19]. One large study, based on the Monitoring Dialysis Outcomes Initiative (MONDO) global database [20], has looked at changes in body composition after dialysis initiation using bioimpedance and found that sex, age, diabetes and initial body composition were all significant predictors [21].

The large range in the prevalence of malnutrition in HD patients from the literature is in part due to the number and diversity of measurements used to characterise the condition. These include: malnutrition scoring tools (such as SGA); volumetric measurements of muscle mass (dual X-ray absorptiometry (DEXA), computed tomography (CT) or magnetic resonance imaging (MRI)); biomarkers (albumin), anthropometric measurements (% weight loss or BMI) and bioimpedance measurements of body composition. To date, there are no universally recommended methods for body composition assessment in haemodialysis patients.

Bioimpedance is an attractive option. It is cheap, quick, non-invasive and able to distinguish fat and lean tissue. The technique measures the impedance of the body to a small applied electric current and uses the impedance data, together with an appropriate model to generate parameters including total body water, intra- and extra-cellular fluid volumes, fat mass and fat free mass (FFM) [22]. Traditional models used to generate FFM estimates are based on an assumption of constant FFM hydration at 73%, meaning that in states of altered hydration such as HD, the models are invalid. In light of this, a model has been developed specifically to enable bioimpedance measurements in renal patients with altered hydration. Chamney et al. proposed a 3-compartment model in which the excess fluid in a patient was considered as a separate compartment, alongside lean tissue and adipose tissue [23]. This introduced the concept of normohydration weight as the weight of the body when lean and adipose tissue compartments have normal fractions of intracellular fluid (ICF) and extracellular fluid (ECF) and also overhydration (OH), which is the fluid volume above or below that of a normohydrated subject, for positive and negative values respectively. The model is based on an assumption that, in health, lean tissue and adipose tissue compartments are normally hydrated and they have fixed proportions of ICF and ECF across all individuals. On this basis, measurement of ECF and ICF can be used with body weight and height to quantify normally-hydrated lean tissue mass, normally-hydrated adipose tissue mass and OH (for model details see Appendix A). This model theoretically allows measurements over a wide range of body composition and fluid status, as long as the assumption about constant fractions of ECF and ICF in the lean and adipose tissue compartments is valid. Measurements of the different components of the model using bioimpedance have been validated against gold standard measurements [24].

Bioimpedance is increasingly being used as part of regular fluid management and has the potential to be incorporated into nutritional management. This audit utilised bioimpedance measurements to investigate whether patients experience significant changes in body composition over the two years after dialysis initiation and, if so, whether any routinely collected parameters or characteristics were associated with these changes.

## 2. Materials and Methods

The Body Composition Monitor (BCM; Fresenius Medical Care, Bad Homburg, Germany) uses multi-frequency bioimpedance spectroscopy to measure resistance and reactance at 50 frequencies, which are used to estimate fluid volumes and in turn lean tissue, adipose tissue, normohydration-weight and overhydration. The renal service of the Leeds Teaching Hospitals NHS Trust, which comprises two hospital—one with inpatient services—and six satellite dialysis units covering much of West Yorkshire introduced body composition monitoring using the BCM in 2009. BCM measurements are used primarily for determination of normohydration-weight of HD patients when setting or reviewing target weights and are saved using the Fluid Management Tool (FMT) software (Fresenius Medical Care, Bad Homburg, Germany). Referrals for measurement are on indication and they are made both pre- and post-dialysis, with post-dialysis measurements being made after sufficient time for redistribution of fluid [25]. This has resulted in the collection of longitudinal data showing changes in lean tissue, adipose tissue and normohydration-weight.

All patients who had more than one bioimpedance result within the two years after dialysis initiation were considered for inclusion in this investigation. The Fluid Management Tool database was interrogated to obtain normohydration-weight, lean tissue and adipose tissue for each measurement. The only exclusion criteria was for measurements made hand-to-hand, rather than the standard hand-to-foot approach, which is usually done for patients with amputated or inaccessible feet or heavily localised oedema. BCM measurements are carried out by trained staff and made in duplicate to verify consistency. All results were based on data collected as part of routine care and extracted from FMT anonymously; as such no ethical approval was required for the study.

There are a great many variables associated with malnutrition in HD, however this study was designed particularly to investigate if any routinely collected variables were associated with measured changes in body composition in HD patients. The variables included in the model, based on previous literature and clinical judgement, were age, initial weight (at HD initiation), gender, ethnicity, diabetes, comorbidity burden, chronic acidosis, chronic inflammation and chronic hyperparathyroidism. Patients were classified as having high comorbidity burden if they scored positive for two or more of the comorbidities recorded on the renal unit database (angina, previous myocardial infarction, coronary artery bypass graft, heart failure, smoker, chronic obstructive pulmonary disease, cerebrovascular disease, diabetes mellitus, malignancy, liver disease, claudication, ischemic/neuropathic ulcers, angioplasty, peripheral vascular disease amputation). Acidosis was indicated by serum bicarbonate below the normal range (22 mmol/L), inflammation was indicated by a serum C-reactive protein (CRP) above normal range (10 mg/L) and hyperparathyroidism was indicated by a serum parathyroid hormone (PTH) greater than 32 pmol/L. A patient was considered as having chronic acidosis, inflammation or hyperparathyroidism where more than half of the routine monthly blood tests over the first two years of dialysis indicated a particular condition.

Subject characteristics were described using mean (standard deviation) or proportions as appropriate. The primary analysis was to describe changes in lean tissue, adipose tissue and normohydration-weight over time using linear mixed-effects models to account for repeated BCM measures on individuals. Subject was taken as the random effect, with time and all the variables associated with malnutrition included as fixed effects. The models were examined with plots of standardised residuals against fitted values to check the assumption of homoscedasticity and Q-Q plots of the residuals to assess normality.

**Figure 1:**
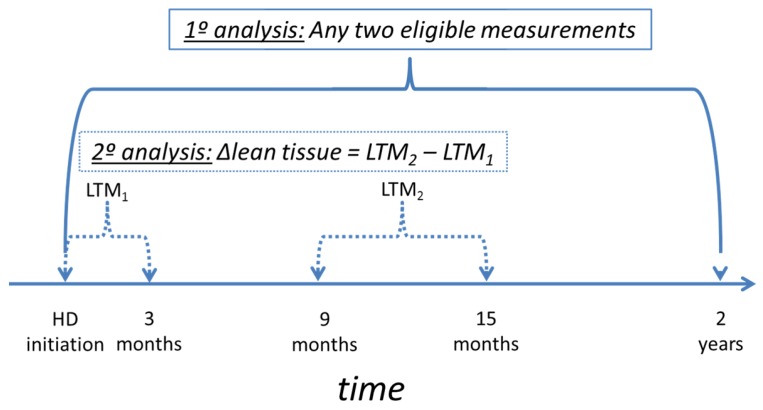
Timeline of data included in primary and secondary analyses. Primary analysis defining body composition changes used data from any patient with more than one Body Composition Monitor (BCM) measurement in the first 2 years of haemodialysis (HD). For secondary analysis of survival, data for calculating the change in lean tissue over the first year of HD, ∆lean tissue came from discrete periods. LTM_1_ is the lean tissue mass from the first BCM measurement in the period 0–3 months. LTM_2_ is the lean tissue mass for the BCM measurement closest to the anniversary of HD initiation, within the 9–15 months window.

Secondary analysis investigated the effect of lean tissue change in the first year of treatment on survival using a Cox-regression model. This analysis was based on a subset of the cohort who had a BCM measurement in the first 3 months after dialysis initiation and again in a window at 9–15 months after dialysis initiation (see Figure 1). Lean tissue change over the first year of HD was defined as the difference between BCM results in these two time periods. Where more than one measurement was available in any given time period, the results closest to HD initiation and 12 months after HD initiation were chosen. Data was censored for end of follow up, transplantation or transferring to another renal centre. Confounding factors were taken as age, chronic acidosis, chronic inflammation, chronic hyperparathyroidism, comorbidity burden and initial weight. The statistical software package ‘R’ version 3.0.2 (R Foundation for Statistical Computing, Vienna, Austria) was used for all analyses.

## 3. Results

### 3.1. Subjects

During the study period, 929 patients started HD within the renal service. Of these, 299 subjects had more than one eligible BCM measurement in the first two years after HD initiation and for these subjects there were a total of 1924 BCM measurements that were included in the regression model. Of these 299 patients, 129 patients had measurements both in the first 3 months of dialysis and again in a window from 9 to 15 months for survival analyses, and this cohort was largely representative of the whole group (Table 1 and Table 2).

### 3.2. Body Composition Changes

The results from the regression models can be seen in Table 3. Over the first two years of haemodialysis, patients typically lost about 0.9 kg of lean tissue and gained about 0.7 kg in adipose tissue, resulting in no significant change in normohydration weight. None of the covariables were significantly associated with any of the body composition compartments, other than patients with lower initial weight tending to gain more adipose tissue than those of higher initial weight.

Mean follow up time for the Cox proportional hazards model was 3 years and 4 months and there were 36 deaths during this period. Multivariate analysis suggested that for every 1 kg gain in lean tissue during the first year of dialysis, there is a 7% reduction in mortality (Table 4). Increasing age and chronic acidosis were also associated with mortality. Univariate analysis showed similar results, although the change in lean tissue was not statistically significant, which is likely to be related to the well-established association between loss of lean tissue and ageing.

## 4. Discussion

These results showed a tendency for patients to lose lean tissue and gain adipose tissue over the first two years of HD, resulting in no change to normohydration-weight. Furthermore, there is a suggestion that these changes in body composition occur across the whole HD population.

Despite known associations between body composition changes and outcomes in HD patients, there are relatively few robust longitudinal studies characterising body composition changes around HD initiation. Use of the MONDO database provided a large dataset of BCM measurements which demonstrated similar changes in body composition to our findings here [21]. Although the choice of predictor variables considered were somewhat different from this study, analysis suggested that female gender, diabetic status and low baseline fat were associated with increases in fat tissue and that diabetes, male gender, high baseline lean tissue and low baseline fat were associated with reductions in lean tissue. The differences with the results presented here could be explained by the relatively small size of our study in comparison and the chance of it being underpowered. John et al. used CT measurements of muscle cross sectional area around HD initiation which also showed similar trends as this data [26]. Interestingly, this study showed that pre-dialysis patients exhibited an even greater rate of muscle loss and that dialysis initiation actually reduced the rate of muscle loss. Other studies were unable to measure notable changes in body composition over 12 months on HD [27,28], but these studies were based on small patient numbers and used a measure of body composition—dual energy X-ray absorptiometry—which is unable to differentiate excess fluid from lean tissue.

These results suggested that the tendency for loss of lean tissue was not confined to patients from any perceived group at risk but was present across the whole population, which has been demonstrated previously with the particular variables from this study [13,26]. There was an association between initial weight and loss of adipose tissue and normally hydrated weight, but this finding could be related to the ‘regression toward the mean’ phenomena or the fact that those of lowest initial weight who lost lean tissue may be less likely to survive and be included in the analysis than those of higher initial weight who lost lean tissue.

The loss of lean tissue as measured by BCM was shown to be associated with mortality, with a 1 kg loss in lean tissue being associated with a 7% reduction in mortality. These results must be viewed with caution as the retrospective nature of the study meant the model was not powered. The inclusion of 7 covariates with only 36 events would generally indicate the potential for over-fitting, although computer modelling has suggested that models with greater than 5 events per covariate are not particularly susceptible to problems [29].

These results support efforts towards monitoring and interventions aimed at preserving lean tissue. Exercise training has been shown to improve muscle mass and function in HD patients [30] and there is growing interest in the provision of appropriate exercise programmes, but the difficulties and barriers to uptake need to be further explored. Bioimpedance is potentially well suited for routine regular monitoring of HD patients, but care must be made when selecting which bioimpedance parameters to use. Previous applications of bioimpedance in this field have used phase angle [27] and lean body mass measured by bioimpedance analysis [31], both of which are confounded by altered fluid status. The parameters from the BCM are independent of fluid status and the high within-subject precision of the test [32] makes the BCM ideal for longitudinal tracking of body composition in clinical practice.

There were some limitations to this study. This was a single centre study and the retrospective nature limited the number of patients that could be included, leading to a relatively small sample size and the possibility that some of the outcomes were not adequately powered. It would also have been interesting to have been able to confirm the changes in body composition using other measurements of nutritional status, such as anthropometric measures or malnutrition scoring tools, but these were not available.

## 5. Conclusions

This study has shown that marked changes in body composition occur in the first two years after HD initiation and these are not confined to elderly, co-morbid patients and those traditionally considered most at risk of wasting. Nutritional monitoring and interventions should be applied across the HD population and this should occur as soon as possible after the initiation of treatment.

## Figures and Tables

**Table 1:** Characteristics of those patients involved in the analysis.

Variable	Whole Group	Survival Analysis Group
*N*	299	129
Age (years)	63 (15)	62 (15)
Gender (% male)	62	60
Ethnicity (% white)	77	76
Diabetes (%)	42	43
High comorbidity burden (%)	44	45
Chronic acidosis (%)	45	41
Chronic inflammation (%)	47	40
Chronic hyperparathyroidism (%)	28	67
eKt/v greater than 1.2 (%)	73	71

Continuous variables are mean values (standard deviation) and categorical values are percentages, unless stated otherwise.

**Table 2:** Characteristics of those patients involved in the primary analysis (whole group) and secondary analysis (survival group).

Variable	Whole Group (Baseline)	Survival Analysis (Baseline)	Survival Analysis (1 Year)
Normohydration weight (kg)	75 (20)	76 (21)	78 (20)
Lean tissue mass (kg)	34 (12)	33 (11)	31 (10)
Adipose tissue mass (kg)	42 (19)	44 (20)	48 (20)

Variables are mean values (standard deviation).

**Table 3:** Mixed results regression model for the change in body composition over the first two years of HD.

Variable	Lean Tissue		Adipose Tissue		Normohydration Weight	
	Value (kg)	95% CI	Value (kg)	95% CI	Value (kg)	95% CI
Time (years)	−0.85 *	−1.1 to −0.60	0.65 *	0.32 to 0.98	0.15	−0.11 to 0.41
Age	−0.01	−0.04 to 0.03	0.02	−0.06 to 0.03	−0.03	−0.06 to 0.01
Sex (male)	−0.56	−1.2 to 0.03	0.37	−0.40 to 1.1	−0.15	−0.76 to 0.47
Ethnicity (White)	0.35	−0.75 to 1.5	0.03	−1.4 to 1.5	0.19	−0.91 to 1.3
Chronic acidosis	0.05	−0.71 to 0.81	0.18	−0.83 to 1.2	0.10	−0.68 to 0.87
Chronic inflammation	−0.34	−1.1 to 0.37	0.44	−0.50 to 1.4	0.27	−0.46 to 1.0
Chronic hyperparathyroidism	−0.02	−0.82 to 0.78	−0.37	−2.2 to 0.50	−0.24	−1.1 to 0.58
Diabetes	0.28	−0.73 to 1.3	−0.98	−2.3 to 0.37	−0.53	−1.6 to 0.48
High comorbidity burden	0.17	−0.86 to 1.2	−0.49	−1.9 to 0.88	−0.15	−1.2 to 0.87
Initial weight (kg)	0.02	−0.01 to 0.04	−0.05 *	−0.08 to −0.02	−0.03 *	−0.06 to −0.01

* indicates *p* < 0.05.

**Table 4:** Cox proportional hazards model to assess the association between some routinely collected parameters linked to malnutrition and risk of death, presenting hazard ratios (HR), 95% confidence intervals (CI) and *p*-values (*p*).

Coefficient	Unadjusted			Adjusted		
	HR	95% CI	*p*	HR	95% CI	*p*
∆lean tissue (kg)	0.95	0.90–1.01	0.08	0.93	0.88–0.98	0.01
Age (years)	1.04	1.01–1.08	0.01	1.04	1.01–1.07	0.02
Chronic acidosis	2.3	1.2–4.7	0.02	2.6	1.9–5.5	0.02
Chronic inflammation	1.8	0.92–3.7	0.08	1.9	0.87–4.1	0.1
Chronic hyperparathyroidism	1.2	0.58–2.4	0.7	1.4	0.66–2.9	0.38
High comorbidity burden	1.3	0.63–2.5	0.5	1.9	0.86–4.1	0.12
Initial weight	1.0	0.98–1.01	0.7	0.99	0.97–1.0	0.3

A total of 128 patients were included in the analysis and there were 36 deaths during follow up and the mean (standard deviation) time between measurements was 305 (50) days.

**Figure A1:**
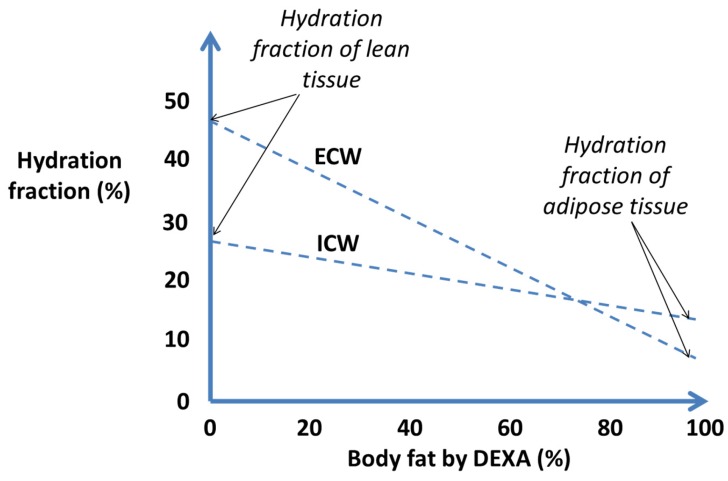
Estimation of the fractional hydration of ECF and ICF in lean and adipose tissue, by extrapolation of regression lines based on dilution based fluid volumes and DEXA body fat. Reproduced from [24].

## Appendix A. Technical Basis and Validation of the BCM Model

The body composition model employed by the BCM [24] is designed specifically to account for the presence of altered fluid status in HD patients. It is centred on the assumption that in health, lean tissue and adipose tissue compartments are normally hydrated and the proportion of ICF and ECF in each compartment does not differ between individuals. On this basis, it is relatively straightforward to show that simply by measuring ECF and ICF and knowing body weight and height, simultaneous equations can allow the quantification of normally-hydrated lean tissue mass, normally-hydrated adipose tissue mass and OH [23].

The values for these fractions were determined experimentally. Simultaneous measurements of dual energy X-ray absorptiometry (DEXA) body fat, ECF with bromide dilution and TBW with deuterium dilution (allowing an estimate of ICF by subtraction of ECF) were made. Percentage body fat was plotted against ECF and ICF and a regression line allowed an estimation of the fractional ECF and ICF at the hypothetical states of 0% and 100% body fat—i.e., the percentage ECF and ICF for lean and adipose tissue (see Figure A1).

The model is commonly used with bioimpedance assessments of ECF and ICF for measuring normo-hydration weight and OH in particular, but also for lean and adipose tissue. There are a number of studies that have attempted to validate the measurements [24,32], but, for normo-hydration weight and OH, this is very difficult as no gold-standard exists. There is a growing body of evidence supporting a link between measurements of OH using this model and outcomes [33,34,35] which further supports the clinical validity of the values from the model. However, it must be acknowledged that all measurement techniques have their limitations and even the criteria methods used in the development of this model have a certain degree of uncertainty which will feed into that of the model. This should be considered for each application of the model.
